# The transcription factor *BpERF1A* enhances drought tolerance in *Betula platyphylla* by activating dehydrin and ROS scavenging through interaction with BpRAV1

**DOI:** 10.1093/hr/uhag133

**Published:** 2026-04-07

**Authors:** Meiqi Zhou, Yuan Zhang, Zhen Miao, Weiyi Bi, Xu Li, Yang Li, Chao Wang

**Affiliations:** State Key Laboratory of Tree Genetics and Breeding, Northeast Forestry University, Harbin 150040, China; State Key Laboratory of Tree Genetics and Breeding, Northeast Forestry University, Harbin 150040, China; State Key Laboratory of Tree Genetics and Breeding, Northeast Forestry University, Harbin 150040, China; State Key Laboratory of Tree Genetics and Breeding, Northeast Forestry University, Harbin 150040, China; State Key Laboratory of Tree Genetics and Breeding, Northeast Forestry University, Harbin 150040, China; State Key Laboratory of Tree Genetics and Breeding, Northeast Forestry University, Harbin 150040, China; State Key Laboratory of Tree Genetics and Breeding, Northeast Forestry University, Harbin 150040, China

## Abstract

Although ERF transcription factors (TFs) play critical roles in abiotic stress tolerance, the molecular mechanisms underlying this role remain incompletely understood. This study identified *BpERF1A* in birch (*Betula platyphylla*) as a drought-responsive TF through co-expression regulatory network analysis. Expression of *BpERF1A* was stalwartly induced by drought stress, and drought treatment markedly boosted its promoter activity. Functional analyses demonstrated that overexpression of *BpERF1A* markedly improved drought tolerance compared with wild-type (WT) birch, whereas its repression increased drought sensitivity. Overexpression lines also exhibited higher antioxidant enzyme activities and proline content relative to WT. Chromatin immunoprecipitation-polymerase chain reaction, yeast one-hybrid and dual-luciferase (dual-LUC) assays confirmed that *BpERF1A* directly binds to G-box elements in the promoters of *BpDHN* (dehydrin) and *BpAOS* (allene oxide synthase), activating their transcription. Furthermore, overexpression of *BpDHN/BpAOS* enhanced drought tolerance and promoted reactive oxygen species (ROS) scavenging in transgenic plants. Protein–protein interaction analysis using bimolecular fluorescence complementation revealed that BpERF1A interacts with BpRAV1 to form a heterodimer, which further enhances BpERF1A binding to the *BpDHN* promoter and its transcriptional activation. Collectively, these findings establish a central role for the *BpERF1A* regulatory module in drought acclimation and highlight its synergistic interaction with BpRAV1, providing an effective strategy to enhance drought tolerance through improved ROS scavenging capacity.

## Introduction

Climate change has exacerbated land degradation and desertification, leaving much of the land suitable for afforestation located in arid, semi-arid, or saline-alkali regions with complex topography, greatly increasing the challenges associated with tree planting [1–3]. Meanwhile, global economic development continues to drive rising demand for high-quality timber for use in construction, paper manufacturing, and other industries [3–5]. Consequently, there is a vital need to establish new tree species that are drought-tolerant, salt-resistant, fast-growing, and capable of producing high-quality wood. Achieving this goal is critical not only for expanding forest coverage and improving afforestation quality but also for supporting ecological restoration efforts and ensuring a sustainable timber supply worldwide.

During their development, plants are constantly exposed to several abiotic stresses, which can induce changes in their morphology and physiology as adaptive strategies to sustain growth [6, 7]. Upon sensing drought stress, plants regulate the synthesis and degradation of chlorophyll (Chl) and other pigments [8], thereby reducing photosynthetic water consumption and protecting leaves from drought-induced damage [9, 10]. Drought also causes cellular dehydration and death, which plants counteract by adjusting their physiological and biochemical processes. These defense mechanisms include responses of the photosynthetic apparatus [11, 12], accumulation of osmoregulatory compounds [13–15], activation of antioxidant enzyme systems [16, 17], and modulation of endogenous plant hormone levels [18–20].

A critical consequence of drought stress is the reactive oxygen species (ROS) excessive accumulation, including superoxide anion (O_2_^−^), hydroxyl radicals (OH^−^), singlet oxygen (^1^O_2_), and hydrogen peroxide (H_2_O_2_) [21–23]. Elevated ROS levels disrupt enzyme and membrane protein conformations, increase membrane permeability and ion leakage, and damage Chl, thereby impairing photosynthesis. This oxidative damage ultimately leads to metabolic dysfunction and, in severe cases, plant death [21, 23]. To mitigate ROS-induced injury, plants activate an intricate antioxidant defense system comprising enzymatic components, such as catalase (CAT), peroxidases (POX), and superoxide dismutase (SOD), as well as non-enzymatic antioxidants, including ascorbic acid, carotenoids, and glutathione [24–26]. These systems act in concert to scavenge ROS and preserve cellular redox homeostasis.

Among the key functional genes mediating drought stress adaptation, dehydrins (DHNs) and allene oxide synthases (AOS) play irreplaceable roles in maintaining cellular integrity and activating stress signaling. DHNs, a subgroup of late embryogenesis abundant (LEA) proteins, are highly hydrophilic and accumulate rapidly under drought-induced cellular dehydration [27, 28]. Their conserved K-segments and Y-segments enable binding to water molecules, ions, and denatured proteins, thereby preventing membrane lipid peroxidation, stabilizing enzyme structures, and reducing ROS-induced oxidative damage, critical for sustaining cell viability under water deficit [27, 29]. AOS, the rate-limiting enzyme in the jasmonic acid (JA) biosynthesis pathway, links drought stress perception to hormone-mediated defense responses [18, 19]. Under drought, AOS catalyzes the conversion of linolenic acid to allene oxide, a key intermediate in JA synthesis; elevated JA levels then trigger a series of adaptive reactions, including stomatal closure to reduce water loss, induction of antioxidant enzyme genes to scavenge ROS, and regulation of stress-responsive transcription factors (TFs) to amplify drought defense networks [18, 20].

Beyond these immediate biochemical defenses, plants initiate a profound genetic reprogramming to enhance drought resilience [30]. This molecular adaptation involves a coordinated regulation of protein synthesis and gene expression, including microRNA (miRNA)-mediated post-transcriptional regulation [31, 32], modulation of functional proteins [33], and synthesis of regulatory proteins [34]. TFs have an essential role in orchestrating these adaptive responses. Several TF families are the drought tolerance key regulators, including AP2/ERF [35], MYB [36], bZIP [37], and NAC (NAM, ATAF1/2, CUC1/2) [38]. Among these, the AP2/ERF superfamily is one of the most widely distributed in plants and has a very critical role in abiotic stress signaling. AP2/ERF TFs boost drought tolerance by promoting antioxidant enzyme activity, modulating endogenous hormone levels, and regulating the drought-defense genes' expression.

Genome-wide studies have identified and characterized the large and diverse ERF gene family in several plant species, such as model plants (e.g. *Arabidopsis thaliana* [39] and rice [40] and economically important crops and trees (e.g. tomato [41], cotton [42], and peanut [43]). These studies collectively highlight the pivotal roles of ERF TFs in regulating several biological processes, including plant growth, abiotic stress responses (particularly drought tolerance), and hormone signaling pathways [18, 19]. Functional analyses in various species have demonstrated that ERFs act through complex regulatory networks, which involve transcriptional regulation of downstream target genes as well as interactions with other regulatory proteins. For example, certain ERFs have been shown to modulate drought sensitivity, enhance antioxidant enzyme activity, improve water-use efficiency (WUE), and influence plant developmental traits [35]. Notably, studies in pear have revealed ERF–MYB interactions that regulate anthocyanin biosynthesis, underscoring the importance of protein–protein interactions in mediating ERF functions [44]. Collectively, these findings provide strong evidence that engineering ERF TFs and their interacting partners represents an auspicious strategy to advance WUE and drought tolerance in crops. Nevertheless, the underlying mechanisms and regulatory networks remain incompletely understood, and many components of these pathways require further elucidation.

*Betula platyphylla* (birch), a fast-growing deciduous tree species in the Betulaceae family, is broadly distributed across northern China and is extensively used for urban greening. It serves as a valuable raw material for furniture and building production, while its sap is rich in nutrients and its bark provides medicinal compounds and pyrolytic gums with therapeutic potential [45]. However, with the increasing prevalence of drought due to global climate change, breeding drought-tolerant birch varieties has become a pressing need.

In our previous work, we constructed drought-responsive transcriptomes of birch, through comparative genomic analysis with *A. thaliana*, identified a differentially expressed gene belonging to the ERF subfamily that was significantly upregulated under drought stress. This gene was designated *BpERF1A*. Building on this discovery and the context outlined above, this study aimed to (i) characterize the expression pattern of *BpERF1A* in various tissues under drought conditions, (ii) elucidate its biological function in drought stress responses, and (iii) analyze its transcriptional regulatory mechanisms, including its downstream target genes and protein interaction partners. Collectively, this work expands our comprehension of the molecular regulatory networks underlying drought resistance in birch and provides a valuable genetic resources and theoretical basis for the breeding of stress-tolerant forest tree species.

## Results

### *BpERF1A* acts as a positive regulator induced by drought stress

**Figure 1:**
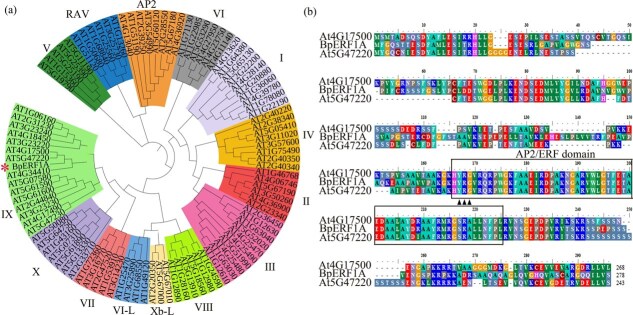
Amino acid sequence alignment and phylogenetic analysis of BpERF1A. (a) Phylogenetic tree of BpERF1A and Arabidopsis ERF family members. The position of BpERF1A is marked with a triangle. Full-length amino acid sequences of *A. thaliana* ERFs were retrieved from the TAIR database and used for tree construction. (b) Multiple sequence alignment of BpERF1A, At4g17500, and At5g47220. The conserved AP2/ERF domain is highlighted with a solid box, and the YRG element is indicated by a triangle. Sequence alignment was done utilizing the ClustalW program. Moreover, the phylogenetic tree was carefully generated with MEGA 11.0 software using 1000 bootstrap replicates.

To recognize drought-responsive TF genes, we analyzed birch transcriptome data generated under progressive drought stress conditions induced by 20% PEG_6000_ treatment. Among the differentially expressed genes, *BpERF1A* (BPChr02G25640) exhibited sustained upregulation, with RPKM values increasing significantly as stress duration progressed (Fig. S1). The coding sequence (CDS) of BpERF1A is 837 bp in length and encodes a 278-amino acid protein. Phylogenetic analysis placed BpERF1A within the ERF IX subgroup (Fig. 1a), confirming its classification as an AP2/ERF TF member. Domain analysis further revealed the existence of a AP2 DNA-binding domain (Fig. 1b), which is highly conserved and critical for its role as a transcriptional regulator.

**Figure 2:**
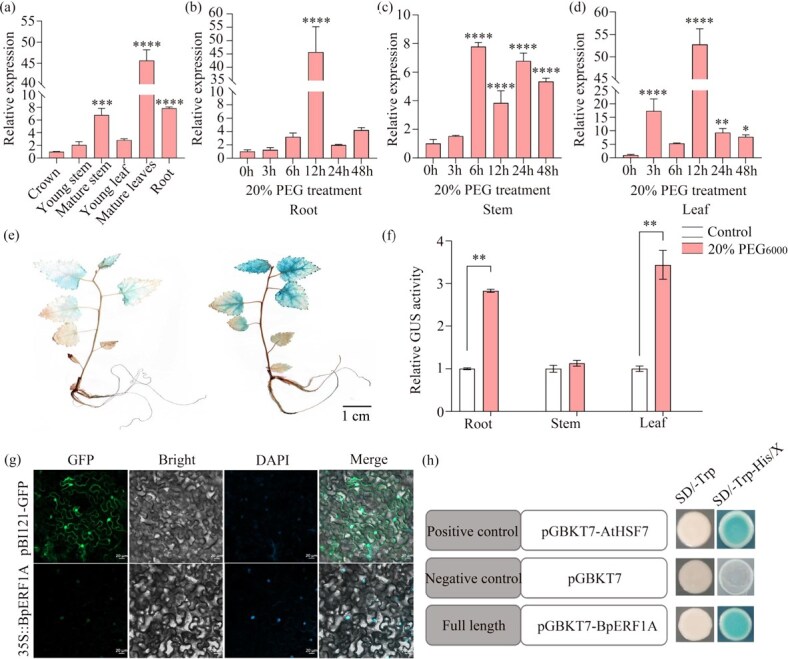
Expression characteristics of *BpERF1A*. (a) Expression levels of the *BpERF1A* in various tissues. (b-d) Expression patterns of *BpERF1A* in roots, stems, and leaves under 20% PEG_6000_ treatment. Data are presented as the mean ± SD (*n* = 6, combining three biological replicates with three technical replicates each). Asterisks denote significant differences versus the control (one-way ANOVA with Tukey’s test) (^*^P < 0.05, ^**^P < 0.01, ^***^P < 0.001, ^****^P < 0.0001). (e) GUS staining analysis. The plants grew on normal MS medium or MS medium supplemented with 20% (v/w) PEG_6000_ for 6 hours, and used for staining. (f) Analysis of the expression of *BpERF1A* by quantifying GUS activity. Control: well-watered plants; PEG: plants treated with 20% PEG_6000_ for 6 hours. (g) Subcellular localization of the BpERF1A protein in onion epidermal cells, with panels showing green fluorescent protein (GFP) fluorescence, bright-field imaging, 4′,6-diamidino-2-phenylindole (DAPI) nuclear staining, and the merged image for spatial reference. (h) Transcriptional activation of *BpERF1A* in the yeast system.

Tissue expression analysis identified that *BpERF1A* was most extremely expressed in old leaves (45.66-fold higher than in terminal buds), followed by roots (7.89-fold) and old stems (6.89-fold) (Fig. 2a). Under drought stress (20% PEG_6000_), reverse transcription-quantitative PCR (RT-qPCR) showed progressive upregulation of *BpERF1A* in roots, stems, and leaves over time, with expression consistently higher in leaves and roots than in stems, peaking at 12 hours with 45.75-fold (roots) and 52.78-fold (leaves) increases compared to untreated controls (Fig. 2b–d). To characterize the drought responsiveness of the *BpERF1A* promoter, we generated transgenic birch lines harboring a *BpERF1A* promoter-driven GUS reporter. Histochemical staining revealed that under normal growth conditions, GUS activity was detectable in both leaves and roots. Upon PEG-induced drought stress, GUS expression was markedly enhanced in these tissues. Quantitative fluorometric GUS assays further confirmed that drought stress significantly induced *BpERF1A* promoter activity in both leaves and roots (Fig. 2e and f). Subcellular localization confirmed that *BpERF1A* resides in the nucleus of *Nicotiana benthamiana* epidermal cells (Fig. 2g). Yeast two-hybrid (Y2H) results demonstrated that both pGBKT7 and *BpERF1A*–pGBKT7 vector plasmids could grow normally on SD/-Trp solid medium plates after transfection into Y2H yeast receptor cells, whereas Y2H yeast receptors transfected with pGBKT7-null could not grow or turn blue on SD/-Trp/-His/X-α-Gal plates. In contrast, the *BpERF1A*–pGBKT7 vector plasmid-transfected yeast cells raised and changed to blue normally on SD/-Trp/-His/X-α-Gal plates, indicating that the *BpERF1A* TF possesses transcriptional activation activity (Fig. 2h). These outcomes suggest that *BpERF1A* is a transcriptional activator induced by drought stress.

### *BpERF1A* enhances drought tolerance in transgenic birch plants

**Figure 3:**
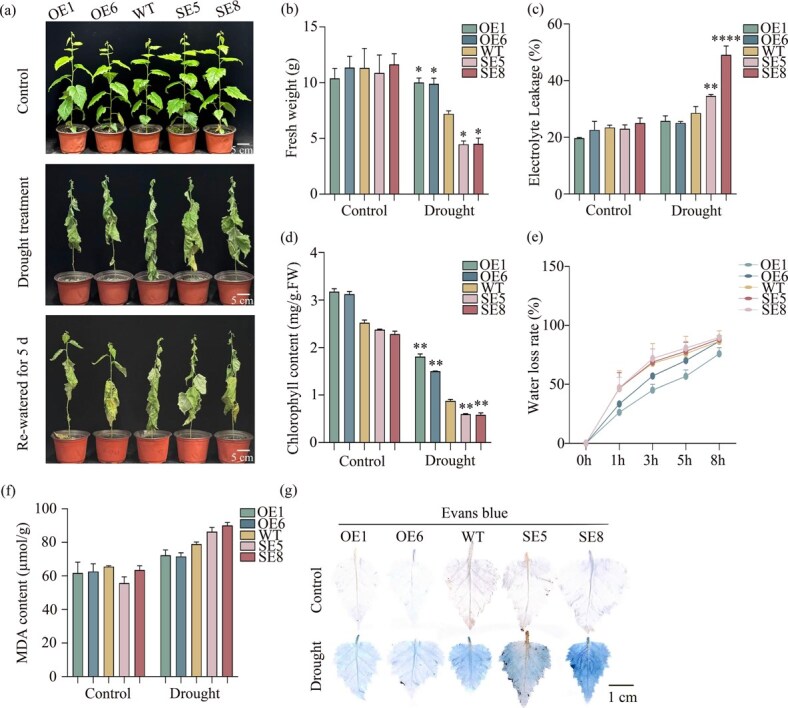
Functional characterization of *BpERF1A* in drought tolerance. (a) Phenotypic comparison between WT and *BpERF1A*-OE birch plants on Days 0 and 15 of drought treatment. (b) Fresh weight measurements of WT, OE, and SE lines. (c) Electrolyte leakage assessment. (d) Chl content analysis. (e) Water loss rate determination. (f) MDA content quantification. (g) Evans blue staining to evaluate cell membrane damage. Birch plants were grown under normal conditions or exposed to drought stress for 15 days without watering, while control plants received regular irrigation. Re-watered: re-watered for 5 days. Data are presented as the mean ± SD (*n* ≥ 3). Asterisks denote significant differences versus the control (one-way ANOVA with Tukey’s test). (^*^P < 0.05, ^**^P < 0.01, ^***^P < 0.001, ^****^P < 0.0001).

To investigate the function of *BpERF1A* under drought stress, transgenic birch plants stably overexpressing (OE) or suppressing (SE) *BpERF1A* were generated using the pFGC5941 vector. RT-qPCR confirmed the positive transgenic lines (Figs S2a and 2b). Two OE lines (OE1 and OE6) showing high and comparable expression levels, along with two suppression (SE) lines with significantly reduced expression (SE5 and SE8), were selected for subsequent analyses. (Fig. S2b). RT-qPCR analysis of its homologous genes in the SE lines showed that their expression was not suppressed (Fig. S2c). Simultaneously, we used the Flag antibody to detect the two selected overexpressed plants. The results showed that the corresponding protein was detected in both OE1 and OE6 (Fig. S2d). Under normal growth conditions, transgenic plants had no phenotypic variances relative to wild-type (WT) plants. However, during drought stress, OE plants displayed significantly enhanced drought tolerance (Fig. 3a). While there were no substantial variances in fresh weight or Chl content among lines under normal conditions, drought treatment resulted in significantly higher Chl content and fresh weight in OE lines than in WT, whereas SE lines showed the lowest values (Fig. 3b and d). Additionally, OE plants had a lower water loss rate than WT plants (Fig. 3e). Similarly, there were no substantial variances in electrolyte leakage or malondialdehyde (MDA) content among all lines under normal growth conditions, but drought stress caused SE lines to display elevated MDA content and electrolyte leakage relative to WT (Fig. 3c and f). Consistently, Evans blue staining revealed darker coloration in SE lines compared with WT, while OE lines exhibited the lightest staining, aligning with the electrolyte leakage results (Fig. 3g). Collectively, these findings indicate that overexpression of *BpERF1A* reduces cell membrane damage and enhances drought tolerance in transgenic birch plants.

### *BpERF1A* enhances ROS scavenging ability

**Figure 4:**
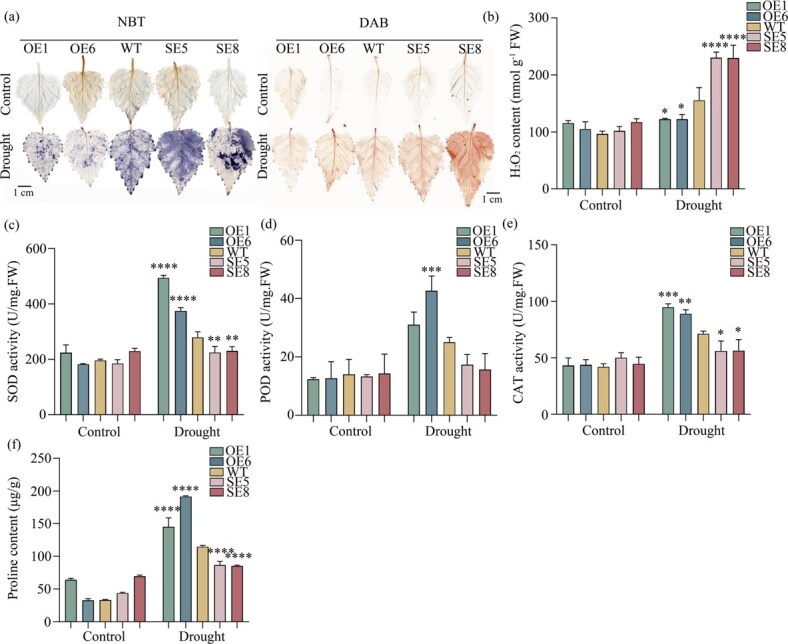
Physiological and histochemical analyses of drought tolerance in *BpERF1A* transgenic lines. (a) NBT and DAB staining showing H_2_O_2_ and O_2_^−^ accumulation in OE, WT, and SE plants under drought and normal conditions. (b) Quantification of H_2_O_2_ content. (c–e) Activities of antioxidant enzymes: POD, SOD, and CAT. (f) Proline content measurement. Data are presented as means ± SD (*n* ≥ 3). Asterisks denote significant differences versus the control (one-way ANOVA with Tukey’s test). (^*^P < 0.05, ^**^P < 0.01, ^***^P < 0.001, ^****^P < 0.0001).

In order to investigate the physiological pathways involved in drought tolerance of *BpERF1A*, we measured drought-related physiological indicators. Nitroblue tetrazolium (NBT) and 3′-diaminobenzidine (DAB) staining showed comparable staining intensity among OE, WT, and SE lines under normal conditions. Nevertheless, under drought stress, OE plants exhibited lighter staining than WT, whereas SE plants displayed the darkest staining, indicating that H_2_O_2_ and O_2_^−^ accumulation was lowest in OE lines (Fig. 4a). Quantitative analysis of H_2_O_2_ content confirmed substantially lower accumulation in OE lines relative to WT, consistent with the staining results (Fig. 4b). We further examined the activities of key antioxidant enzymes (e.g. SOD, POD, and CAT) in drought-stressed plants. OE lines showed substantially boosted activities of these enzymes relative to WT and SE lines (Fig. 4c–e), confirming *BpERF1A*'s role in enhancing ROS scavenging capacity. Because proline accumulation is an adaptive response to drought, we also quantified its content. OE lines accumulated significantly more proline than WT and SE lines, with SE showing the lowest levels (Fig. 4f). Collectively, these findings demonstrate that *BpERF1A* mitigates drought-induced cellular damage by boosting antioxidant enzyme activities and reducing ROS accumulation.

### BpERF1A directly regulates the transcription of *BpDHN* and *BpAOS*

**Figure 5:**
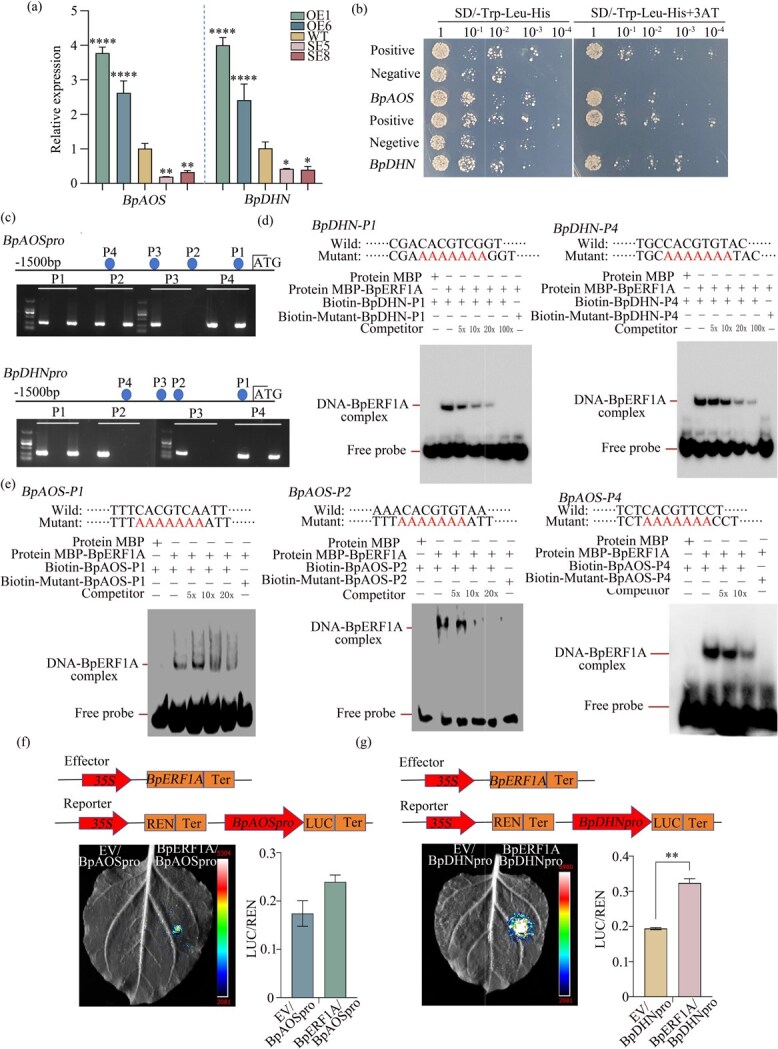
BpERF1A directly regulates *BpDHN* and *BpAOS* transcription by binding to their promoters. (a) Relative expression levels of *BpDHN* and *BpAOS* in *BpERF1A*-OE and SE lines compared with WT plants. Data are shown as means ± SD from three biological replicates. (b) Y1H assay confirming BpERF1A binding to the promoters of *BpDHN* and *BpAOS*. Positive control: p53-HIS2/pGADT7-Rec2-p53; negative control: pGADT7-Rec2-*BpERF1A*/p53-HIS2. 3AT: 50 mM. (c) ChIP-PCR verifying in vivo binding of *BpERF1A* to *BpDHN* and *BpAOS* promoters. Promoter regions (−1500 to −1 bp) were divided into four fragments (P1–P4). ‘Input’ represents total chromatin prior to immunoprecipitation; ‘Mock’ represents mock immunoprecipitation without antibody; ‘ChIP+’ represents immunoprecipitation with anti-Flag antibody. (d, e) EMSA confirmed that the BpERF1A protein also directly binds to G-box-containing probes derived from both the *BpAOS* and *BpDHN* promoter fragment. Biotin-labeled probe incubated with MBP alone was used as a negative control. Biotin-labeled probe incubated with BpERF1A-MBP protein was tested. Promoter fragments were labeled with biotin. Competitive probes of 5×, 10×, 20×, and 100× (lack of biotin label) were used. Wild, core sequence; Mutant, mutated sequence; P, probe. (f, g) dual-LUC reporter assay showing that transient coexpression of BpERF1A with *BpAOS* (e) and *BpDHN* (f) promoter-LUC constructs in *Nicotiana benthamiana* leaves significantly enhanced relative LUC activity (LUC/REN) compared with control. Data are presented as means ± SD (*n* ≥ 3). Asterisks denote significant differences versus the control (one-way ANOVA with Tukey's test). (^*^P < 0.05, ^**^P < 0.01, ^***^P < 0.001, ^****^P < 0.0001).

In order to elucidate the downstream regulatory mechanism of BpERF1A, candidate target genes were screened based on a pre-constructed birch drought-response regulatory network [46]. Two putative drought-related target genes, *BpDHN* and *BpAOS*, were selected for validation. Interrogation of our transcriptome data revealed that the expression patterns of *BpDHN* and *BpAOS*, as measured by FPKM values, were highly consistent with that of *BpERF1A* (Fig. S3a and b). Subsequently, RT-qPCR analysis revealed that both genes were strongly induced by drought stress (Fig. S3c and d). Subsequently, RT-qPCR was used to examine their expression in *BpERF1A* transgenic plants, showing significantly elevated expression of *BpDHN* and *BpAOS* in OE lines relative to WT, whereas expression was reduced in SE lines (Fig. 5a), indicating that *BpERF1A* positively regulates their transcription. To verify direct binding, Yeast one-hybrid (Y1H) assays demonstrated that BpERF1A could interact with the promoter regions of *BpDHN* and *BpAOS* (Fig. 5b). Chromatin immunoprecipitation PCR (ChIP-PCR) further confirmed in vivo binding, as truncated promoter fragments of *BpDHN* (p1, p4) and *BpAOS* (p1, p2, p4) were significantly enriched following immunoprecipitation of BpERF1A–Flag protein (Fig. 5c). To further validate the specificity of BpERF1A binding to these promoter fragments, we performed extended EMSA and Y1H assays. First, EMSA confirmed that the recombinant BpERF1A–MBP fusion protein directly bound to biotin-labeled probes containing the G-box motif from the *BpAOS* and *BpDHN* promoters. The binding specificity was further validated by competition assays, addition of excess unlabeled WT probes effectively competed away the shifted bands, whereas mutation of the G-box core sequence completely abolished binding (Fig. 5d and e). Second, comprehensive Y1H assays were conducted using all four truncated fragments (P1-P4) of the BpDHN and BpAOS promoters as bait. The results showed that BpERF1A specifically interacted with the BpDHN–P1/P4 and BpAOS–P1/P2/P4 fragments, consistent with the ChIP-PCR enrichment profile, but not with the other fragments (Fig. S4a and b). Finally, dual-LUC reporter assays in tobacco leaves revealed that coexpression of BpERF1A with *BpDHN* or *BpAOS* promoter-driven LUC constructs significantly enhanced relative LUC activity (LUC/REN) compared with controls (Fig. 5f and g). Collectively, these results indicate that BpERF1A directly binds to the G-box elements in the promoters of *BpDHN* and *BpAOS*, activating their transcription and thereby contributing to drought stress tolerance.

### Enhancement of drought tolerance in transgenic birch by *BpDHN*

**Figure 6:**
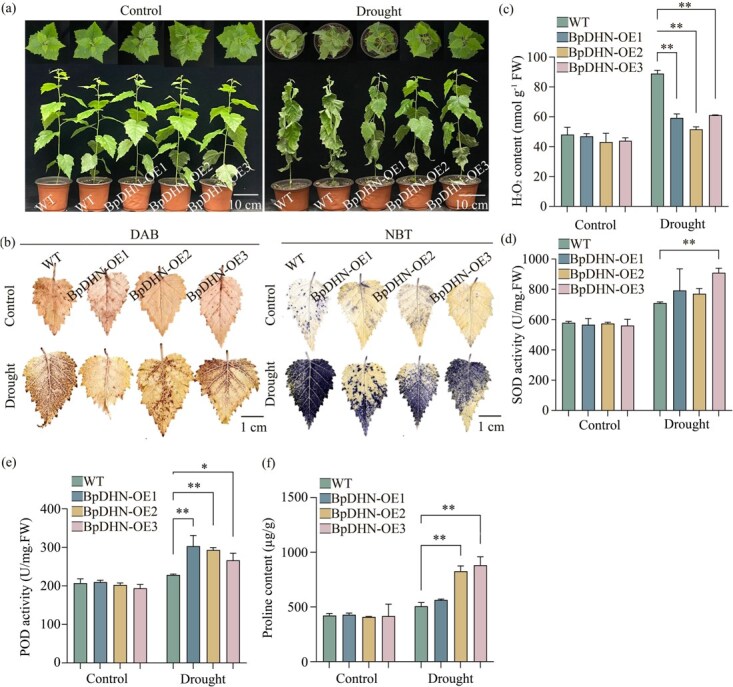
Functional characterization of *BpDHN* in conferring drought tolerance. Phenotypic comparison of WT and *BpDHN*-OE plants at 0 and 15 days of drought treatment. (b) DAB and NBT staining of leaves to visualize H_2_O_2_ and O_2_^−^ accumulation, respectively. (c) Quantification of H_2_O_2_ content. (d) SOD activity analysis. (e) POD activity analysis. (f) Proline content measurement. Birch growth phenotypes were monitored under normal conditions for 90 days. Control plants were regularly watered, whereas drought-stressed plants were exposed for 15 days to water scarcity. Each experimental replicate included 5–10 plantlets. Data are presented as means ± SD (*n* ≥ 3). Asterisks denote significant differences versus the control (one-way ANOVA with Tukey's test). (^*^P < 0.05, ^**^P < 0.01, ^***^P < 0.001, ^****^P < 0.0001).

Drought stress induces *BpDHN* expression. To further characterize the function of *BpDHN* in drought response, a *BpDHN* overexpression vector was constructed, which was then transformed into birch, generating transgenic plants OE *BpDHN* (*BpDHN*-OE) (Fig. S5). We then assessed physiological and biochemical indices of WT and *BpDHN*-OE plants under drought stress. No significant phenotypic variances were noted between *BpDHN*-OE and WT plants under normal conditions. However, when subjected to drought stress, the transgenic plants exhibited enhanced drought tolerance, with less leaf wilting and improved overall growth compared with WT (Fig. 6a). DAB staining revealed darker leaf staining in WT plants, indicating higher H_2_O_2_ accumulation, whereas *BpDHN*-OE plants displayed lighter staining, suggesting reduced H_2_O_2_ levels (Fig. 6c). Similarly, NBT staining demonstrated higher O_2_^−^ accumulation in WT leaves than in *BpDHN*-OE leaves under drought stress (Fig. 6b).

We further measured antioxidant enzyme activities, including SOD and POD. Under drought stress, both enzymes showed substantially higher activities in *BpDHN*-OE plants relative to WT (Fig. 6d and e), suggesting that transgenic plants possessed robust ROS-scavenging capacity, thereby alleviating cellular oxidative damage. Additionally, proline content was significantly higher in *BpDHN*-OE plants relative to WT (Fig. 6f). Together, these findings confirm that overexpression of *BpDHN* markedly raises drought tolerance in birch, providing effective protection for growth and development under drought conditions.

### *BpAOS* overexpression enhances antioxidant capacity and mitigates drought-induced oxidative damage

To validate the physiological function of *BpAOS* in drought response, we employed an Agrobacterium-mediated transient transformation system previously established in our laboratory for birch [47]. The recombinant vector 35S::*BpAOS*–GFP was transiently introduced into birch plants, with the 35S::GFP empty vector serving as the control. Total RNA was extracted from the transiently transformed *BpAOS*-OE and control plants. qRT-PCR analysis confirmed a significant upregulation of *BpAOS* expression in the OE plants compared to the control (Fig. S6).

Under drought stress, *BpAOS*-OE plants accumulated significantly lower levels of H₂O₂ than the control. Correspondingly, compared to the control，the activity of key antioxidant enzymes, POD and CAT, were markedly elevated in the *BpAOS*-OE plants under drought conditions. Furthermore, the content of MDA, a reliable marker of membrane lipid peroxidation and oxidative damage, was significantly reduced in the *BpAOS*-OE plants compared to the control under drought stress (Fig. S7). Collectively, these results indicate that overexpression of *BpAOS* enhances ROS scavenging capacity, alleviates oxidative stress, and protects cellular membranes from drought-induced damage.

### BpERF1A and BpRAV1 co-regulate *BpDHN* expression

**Figure 7:**
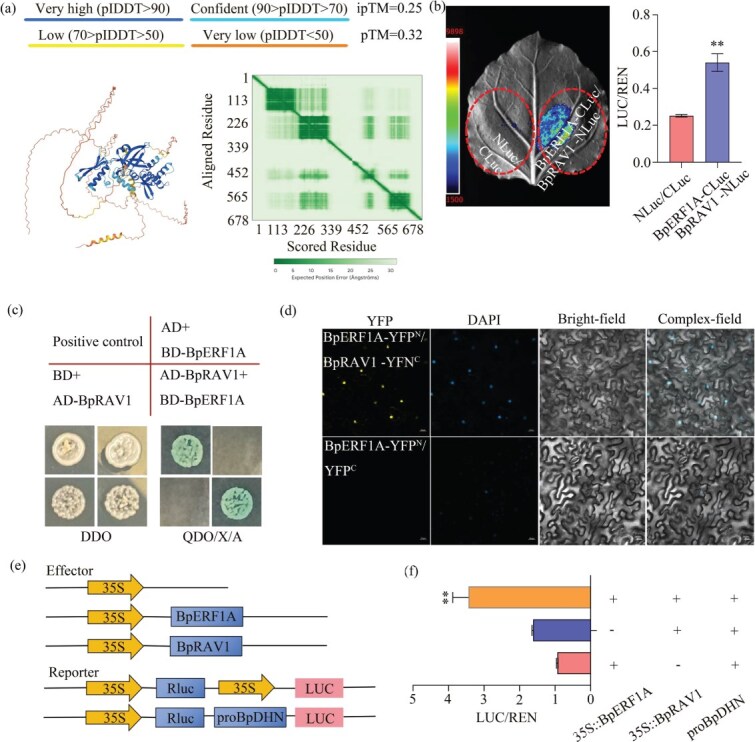
illustrates the interaction between BpERF1A and BpRAV1 and their cooperative role in enhancing *BpDHN* transactivation. (a) Structural modeling of BpERF1A and BpRAV1 predicted by AlphaFold3, indicating a high-confidence interaction (iPTM+PTM > 0.5). (b) Firefly LCI assay confirming the physical interaction between BpERF1A and BpRAV1 through Agrobacterium-mediated co-expression in *N. benthamiana* leaves, with luminescence signals observed 48 hours after infiltration. (c) Y2H analysis of the interactions between BpERF1A and BpRAV1. The positive control was pGADT7-T + pGBKT7-p53. (d) BiFC assay demonstrating that BpERF1A physically interacts with BpRAV1 *in vivo*, forming heterodimers. (e) dual-LUC reporter assay showing that BpERF1A and BpRAV1 individually activate the *BpDHN* promoter, with co-expression resulting in significantly enhanced relative promoter activity (LUC/REN). Dual-LUC reporter assay showing that BpERF1A and BpRAV1 individually activate the *BpDHN* promoter, with co-expression resulting in significantly enhanced relative promoter activity (LUC/REN). Data are presented as mean ± SD (*n* = 6 biological replicates). Statistical significance was determined by Student's *t*-test (^*^P < 0.05, ^**^P < 0.01).

Protein interaction network analysis of birch AP2/ERF family members, predicted using the STRING database, revealed several key topological hubs with high connectivity degrees: BPChr13G24680 (ERF13, degree = 5), BPChr13G08902 (ERF5, degree = 5), BPChr09G29865 (RAV1, degree = 6), BPChr06G02264 (F25G13, degree = 7), and BPChr06G11136 (AP2, degree = 12) (Fig. S8). These connectivity patterns highlight the central role of BpRAV1 within the protein interaction network. Notably, BpRAV1 was predicted as a putative interaction partner of BpERF1A (Fig. S5). To investigate whether *BpRAV1* is involved in the drought response, we examined its expression pattern under drought conditions. RT-qPCR analysis revealed that *BpRAV1* expression was significantly induced by 20% PEG_6000_ treatment, with an expression pattern similar to that of *BpERF1A* (Fig. S9). Therefore, it is speculated that BpRAV1 may work in synergy with BpERF1A in response to drought stress. Further supporting its functional significance, BpRAV1 was also predicted to interact with multiple ERF and DREB subfamily members, suggesting its pivotal role within the AP2/ERF regulatory network. Structural modeling using AlphaFold3 provided additional mechanistic insight, predicting potential physical interactions between BpRAV1 and BpERF1A with high confidence (iPTM+PTM > 0.5) (Fig. 7a).

To experimentally validate this interaction, we first employed firefly LCI assays, which confirmed a interaction between BpERF1A and BpRAV1 (Fig. 7b). This interaction was further corroborated by Y2H assays (Fig. 7c). Finally, BiFC assays in plant cells showed strong fluorescence signals, visually confirming that BpRAV1 and BpERF1A interact and can form heterodimers (Fig. 7d). Collectively, results from these complementary approaches consistently demonstrate a specific interaction between BpERF1A and BpRAV1. We next explored whether BpERF1A and BpRAV1 function cooperatively to regulate *BpDHN* expression. Using an in vivo luciferase reporter system, we found that both BpERF1A and BpRAV1 significantly activated the *BpDHN* promoter individually, whereas co-expression of both proteins resulted in a markedly stronger activation (Fig. 7e and f). Collectively, these findings demonstrate that BpERF1A and BpRAV1 synergistically regulate *BpDHN* expression, with their simultaneous presence enhancing transcriptional activation and contributing to drought stress response.

## Discussion

In this study, a differentially expressed gene was identified in the transcriptome of birch subjected to drought stress, based on the construction of multiple stress treatment-related transcriptomes using birch as test material in a previous study. Sequence analysis revealed that this gene contained an AP2 structural domain belonging to the ERF subfamily, and it was designated as BpERF1A based on homology comparison with *A. thaliana*. Identification of drought-responsive TFs is critical for elucidating the adaptive mechanisms of plants under water-deficit conditions. Here, we demonstrated that the ERF TF *BpERF1A* in birch acts as a drought resistance positive regulator by enhancing ROS scavenging, promoting osmolyte accumulation, and directly activating downstream stress-responsive genes. These findings offer novel insights into the molecular mechanisms of ERF-mediated drought tolerance in woody plants.

### *BpERF1A* as a drought-responsive transcriptional activator

Genome-wide analyses of ERFs have been systematically conducted in several species, such as 145 AtERFs in *Arabidopsis* [39], 139 OsERFs in rice [40], and 256 GmERFs in soybean [48]. ERF proteins are marked by a conserved AP2/ERF structural domain that specifically binds to the promoters of target genes through β-sheet folding to the GCC-box or DRE elements [49, 50]. Recent investigations have revealed that ERF TFs are key participants in plant responses to abiotic stresses, functioning within positive or negative regulatory networks under various adversities, including drought, salinity, low temperature, and pathogen attack [51–53]. Under drought stress, ERF family members frequently activate downstream stress-related genes through transcriptional cascades. For example, the AP2/ERF TF *PtoERF15* in *Populus* confers drought tolerance through JA-mediated signaling [18], while TaERF3 interacts with GCC-box cis-elements in the promoters of seven stress-associated genes, thereby positively regulating wheat adaptation to salt and drought stress through transcriptional activation of its targets [54]. Considering the functional diversity of ERF proteins across species, our research team selected ERFs with high RPKM values under drought stress based on transcriptome data generated in the early stages of this study. Phylogenetic tree analysis and multiple sequence alignment identified BpERF1A as a member of the ERF IX subgroup, containing a conserved AP2 structural domain (Fig. 1a and b), which is essential for DNA binding. Further experiments demonstrated that *BpERF1A* expression was significantly induced by drought stress (Fig. 2b–d), and that GUS activity driven by the *BpERF1A* promoter was strongly enhanced under PEG_6000_ treatment (Fig. 2e and f). Transgenic birch lines OE *BpERF1A* displayed significantly enhanced drought tolerance, whereas repression of *BpERF1A* expression markedly reduced drought tolerance (Fig. 3). Our findings on *BpERF1A* align with the conserved role of ERF TFs in drought tolerance across plant species. For example, overexpression of *OsERF71* alters root architecture and enhances drought resistance in *rice* [55]. Similarly, in *maize*, *ZmERF21* promotes drought tolerance by directly regulating hormone signaling and stress-responsive genes [56]. In *tobacco*, drought resistance is conferred by NtERF172 through its modification of *NtCAT* [57]. These cross-species parallels underscore the pivotal function of ERF TFs in adaptation to water deficit. Collectively, these findings establish *BpERF1A* as a critical drought stress responses positive regulator in birch.

### *BpERF1A* can efficiently eliminate the buildup of ROS

Drought stress leads to excessive accumulation of ROS, including O_2_^−^ and H_2_O_2_, by disrupting the redox equilibrium in plant cells, thereby triggering oxidative stress manifestations such as membrane lipid peroxidation, protein denaturation, and DNA damage [58, 59]. In this study, We observed that the OE of *BpERF1A* did not show significant differences in electrolyte leakage rate and MDA content compared to the WT (Fig. 2). We hypothesize that this phenomenon may be due to the hierarchical activation of the plant defense system during the early stages of stress. Antioxidant enzyme activities (SOD, POD, CAT) and osmoprotectants are likely prioritized, effectively delaying the onset of membrane lipid peroxidation [22]. However, *BpERF1A*-OE lines effectively mitigated ROS accumulation by markedly enhancing the activities of POD, SOD, and CAT under drought conditions, which consequently reduced the buildup of H_2_O_2_ and O_2_^−^ (Fig. 4a–e). These results are consistent with the classical antioxidant defense mechanisms in plants, suggesting that *BpERF1A* enhances ROS scavenging capacity and alleviates oxidative damage by indirectly or directly modulating the expression of antioxidant enzyme-encoding genes.

In addition, proline, a key osmoregulatory compound, not only maintains cellular osmotic balance under drought stress and protects membrane structure but also acts as a ROS scavenger by directly eliminating hydroxyl radicals (•OH), thereby providing dual protection [60]. We observed a substantial rise in proline content in OE lines compared with WT (Fig. 4f), showing that *BpERF1A* may enhance drought tolerance by strongly activating the proline biosynthesis pathway. This result is in line with several previous reports on *TaERF87* in wheat [50] and *OsProDH* in rice [61], indicating that precise regulation of proline metabolism is one of the critical strategies for plant drought adaptation. Notably, the dual regulatory role of *BpERF1A* in promoting both ROS scavenging and proline accumulation may represent an ‘antioxidant-osmotic synergistic’ defense network that has evolved in woody plants to cope with prolonged water-deficit stress.

### BpER1A binds to G-box to regulate target gene expression

DHNs, a major subgroup of the LEA protein family, play a critical cytoprotective role in plant responses to drought stress [27]. These highly hydrophilic proteins bind water, ions, and biomolecules via their conserved K-, S-, and Y-fragment domains, thereby preventing protein denaturation, membrane structural collapse, and ROS bursts triggered by cellular dehydration [28]. Our analysis illustrated that *BpDHN* expression was substantially induced under drought stress (Fig. S3), consistent with the expression pattern of *BpERF1A*. We further demonstrated that the *BpERF1A* protein specifically recognizes and binds to the G-box elements in the promoters of *BpDHN* and *BpAOS*, thereby promoting their transcription, as confirmed by Y1H, EMSA, and LUC assays (Fig. 5). Importantly, overexpression of *BpDHN* in transgenic plants significantly increased SOD, POD, and CAT activities (Fig. 6), indicating that DHNs enhance drought tolerance by maintaining cellular osmotic pressure and protecting membrane integrity. This finding corroborates previous studies, such as the overexpression of SiLEA5 in *Solanum lycopersicum*, which reduced electrolyte leakage and MDA levels while increasing antioxidant enzyme activities and osmolyte content, ultimately reducing oxidative damage and improving drought tolerance [29].

Additionally, we identified *BpAOS*, a key enzyme in JA biosynthesis, as a direct target of BpERF1A. JA is a well-established phytohormone that plays a critical role in mediating plant responses to abiotic stresses, including drought [62]. It orchestrates a wide range of defensive processes, such as the induction of antioxidant enzymes, stomatal regulation, and the synthesis of protective metabolites. The direct transcriptional activation of *BpAOS* by BpERF1A suggests that the enhanced drought tolerance in *BpERF1A-OE* plants may be partly attributed to an elevated JA level (Fig. S10), which could potentiate the antioxidant system and reinforce stress signaling. This is consistent with studies in *poplar*, where *PtoERF15* enhances drought tolerance via JA-mediated signaling [18]. Therefore, we propose that BpAOS acts as a functional bridge, linking the BpERF1A transcriptional module to JA-mediated drought adaptation, thereby adding a hormonal dimension to the BpERF1A-regulated defense network. Collectively, our results establish a complete signaling pathway, *BpERF1A*–*BpDHN*/*BpAOS*-physiological drought response, thereby providing new mechanistic insights into ERF-mediated drought tolerance in woody plants.

### BpERF1A and BpRAV1 interact to enhance *BpDHN* gene expression

To further elucidate the molecular mechanism underlying BpERF1A-mediated drought resistance, we identified and validated its interaction with the TF BpRAV1. Notably, *BpRAV1* expression itself was rapidly induced by drought stress (Fig. S9), suggesting its co-deployment with *BpERF1A* under water deficit. This finding is consistent with the role of RAV, which contain both AP2 and B3 DNA-binding domains as important regulators of plant stress responses. For instance, in *rice*, overexpression of *OsRAV2* enhances drought resistance by modulating proline, MDA, and H_2_O_2_ levels and upregulating ABA signaling genes [63]. Similarly, in *tobacco*, *NtERF172* improves drought tolerance and WUE by reducing stomatal aperture and transpiration rate [57].

We experimentally confirmed the nuclear interaction between BpERF1A and BpRAV1 through BiFC and Y2H assays (Fig. 7). Critically, their co-expression synergistically enhanced *BpDHN* promoter activity, suggesting that heterodimer formation increases transcriptional efficiency.

Interestingly, our investigation into other species reveals that the documented binding of SlRAV2 to the promoter of *SlERF5* in tomato establishes a regulatory hierarchical relationship [64], wherein a RAV protein transcriptionally controls an ERF gene. In contrast, our data demonstrate a direct protein–protein partnership between BpERF1A and BpRAV1. This partnership operates at the same regulatory level, enabling their synergistic action on the promoters of *BpDHN*, rather than constituting a linear cascade. This molecular mechanism of co-activation may permit more precise and effective transcriptional reprogramming under stress. The functional significance of the BpERF1A–BpRAV1 module is likely accentuated within the context of woody perennial biology. We propose that the BpERF1A–BpRAV1 relationship is not merely an example of conserved TF interaction, but is specifically adapted to reinforce a core cellular protection strategy essential for woody plants.

**Figure 8:**
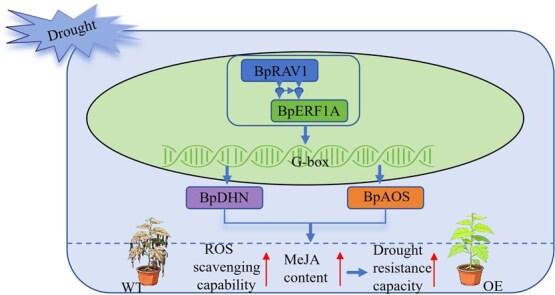
Schematic diagram of *BpERF1A* regulating the drought resistance mechanism of birch.

In conclusion, our study uncovers a molecular regulatory module consisting of BpERF1A, BpRAV1, and their target genes BpDHN/BpAOS. Under drought stress, BpERF1A interacts with BpRAV1 to synergistically activate BpDHN transcription, thereby enhancing ROS-scavenging capacity and ultimately improving drought tolerance in birch (Fig. 8). These findings offer fresh insights into the transcriptional networks orchestrating drought resistance in woody plants and may inform future strategies for genetic improvement of forest species.

## Materials and methods

### Plant material and stress treatments

Birch seedlings grown in a greenhouse for 30 days were used as experimental material and irrigated with a 20% (w/v) PEG_6000_ solution. Tissue samples from three seedlings were randomly collected at 3, 6, 12, 24, and 48 hours following treatment. These samples were subsequently used to analyze the expression patterns of each gene under drought stress.

For phenotypic analysis of transgenic material, WT and transgenic birch trees were transplanted into soil and cultivated for 3 months before being subjected to a 15 days dehydration treatment. Normally, watered plants served as controls. Each line consisted of at least nine seedlings. These plants were then used for phenotypic observations and physiological analyses.

### Phylogenetic analysis and multiple sequence alignment

MEGA 11 software was used to perform multiple sequence alignment between *BpERF1A* and protein sequences from the Arabidopsis AP2/ERF gene family. The resulting alignment files were analyzed using the ‘Find Best DNA/Protein Models’ function, and an evolutionary tree was established utilizing the neighbor-joining (NJ) method with 1000 bootstrap replicates. The best-fit model (JTT + G) was applied, while other parameters were set to default. The resulting tree was exported in Newick (.nwk) format and visualized using the iTOL website. Additionally, protein–protein interaction networks of the AP2/ERF family members were predicted utilizing the STRING database (https://string-db.org/), with *A. thaliana* selected as the reference species, the confidence score threshold set to 0.4, and other parameters maintained at default settings.

### RNA extraction and RT-qPCR assay

Total RNA was extracted from birch samples using the CTAB method, and its integrity and purity were assessed to ensure suitability for downstream experiments. Complementary DNA (cDNA) was subsequently synthesized from the extracted RNA using the TransScript® One-Step gDNA Removal and cDNA Synthesis SuperMix. The resulting cDNA served as the template for RT-qPCR, which was carried out utilizing TransStart® Top Green qPCR SuperMix on a qTower 2.2 system (Analytik Jena AG, Jena, Germany; Cat. 549). The reactions were performed according to the manufacturer's instructions, including both thermal cycling parameters and reaction mixture composition. Relative gene expression levels were calculated using the 2^−ΔΔCt^ method, with ubiquitin (UBQ; FG065618) and tubulin (TU; FG067376) serving as internal reference genes for normalization. Each analysis was conducted with three biological replicates. The primer sequences utilized for the assays are provided in Table S1 (online Supplementary Material).

### GUS staining and quantification of GUS activity

To assess the drought responsiveness of the *BpERF1A* promoter in stable transgenic plants, we generated birch lines harboring a *BpERF1Apro*::*GUS* reporter construct. A 1.5-kb promoter fragment upstream of the *BpERF1A* start codon was cloned into the binary vector pCAMBIA1301 upstream of the β-glucuronidase (GUS) gene. This construct was introduced into birch via *Agrobacterium* tumefaciens-mediated stable root transformation. For histochemical GUS staining, tissues from 4-week-old transgenic seedlings (either untreated or subjected to 20% PEG_6000_ induced drought stress for 12 hours) were harvested. Samples were vacuum-infiltrated and incubated in GUS staining solution [1 mM 5-bromo-4-chloro-3-indolyl-β-d-glucuronide (X-Gluc), 50 mM sodium phosphate buffer (pH 7.0), 0.5 mM potassium ferrocyanide, 0.5 mM potassium ferricyanide, 0.1% (v/v) Triton X-100] at 37°C overnight. Chl was subsequently removed by destaining in 70% (v/v) ethanol. The Gus enzyme activity was determined by measuring the content of 4-methylumbelliferone according to the method of the kit (Shanghai MLBIO, ml064287).

### Vector construction and plant transformation

The *BpERF1A* gene sequence was analyzed using the enzyme cleavage site statistics tool (https://www.detaibio.com/sms2/restsummary.html), and suitable restriction sites available on the pBI121–6 × Flag vector were selected. The *BpERF1A* CDS was then cloned into the pBI121–6 × Flag vector by introducing XbaI/XmaI restriction sites to generate the fusion expression vector *BpERF1A*–pBI121–6 × Flag. For RNA interference (RNAi)-mediated silencing of BpERF1A, we employed the pFGC5941 vector utilizing an intron-spliced hairpin RNA (ihpRNA) strategy. To minimize potential off-target effects, a 300-bp fragment with high specificity, derived from the *BpERF1A* coding sequence, was selected for hairpin construction. Specifically, this fragment was cloned in sense and antisense orientations, separated by the CHSA intron, into the vector via defined restriction sites (*Nco* I/*Asc* I sites at the 5′ end and *Xba* I/*Pac* I sites at the 3′ end). All resulting vectors were transformed into *A. tumefaciens* strain GV3101, and Agrobacterium-mediated stable root transformation was performed to create transgenic birch lines.

Birch roots grown for 30 days were used as recipient material, and Agrobacterium with an OD_600_ of 0.6 was used to infect the birch roots in liquid 1/2 MS medium containing 100 μM AS and 5 μL Tween-20. Roots were cut into 1-cm segments, soaked in the infiltration solution for 10 minutes, and incubated in the dark for 48 hours. Subsequently, the root segments were transferred to screening medium until callus tissue grew at the incision site. Once the healing tissue reached a certain size, it was transferred to differentiation medium with the appropriate selection agent until resistant seedlings developed. The method of transient infection of birch by was carried out as described by predecessors [47].

### Localization of *BpERF1A*

The CDS sequence of *BpERF1A*, with the stop codon removed, was inserted into the pBI121–GFP vector using the Seamless Cloning Kit and fused in-frame to the N-terminal end of GFP to construct the *BpERF1A*–pBI121–GFP fusion expression vector for subcellular localization analysis. Agrobacterium cultures containing either *BpERF1A*–pBI121–GFP or the empty vector were used to infiltrate *Nicotiana benthamiana* leaves overnight [65]. GFP signals were observed at 488 nm using a laser confocal microscope (Carl Zeiss, Oberkochen, Germany), and the nucleus was visualized using DAPI staining.

### Determination of transcriptional activation activity of *BpERF1A*

The full-length CDS of *BpERF1A* was fused to the *GAL4* DNA-binding domain in the pGBKT7 vector. The resulting *BpERF1A*–*pGBKT7* construct was introduced into Y2H yeast receptor cells. After incubation on SD/-Trp medium at 30°C for 3 days, positively growing clones were selected and transferred to SD/-Trp-His/X-α-gal medium for further screening. To assess whether *BpERF1A* possesses self-activating activity, the empty *pGBKT7* vector was introduced into Y2H yeast receptor cells as a negative control, while the reported *AtHSF* gene served as a positive control.

### Western blot analysis

Total protein was extracted from leaves of WT and *BpERF1A*-OE transgenic birch plants using a plant protein extraction kit (P0013B, Biyuntian). Proteins were separated by 10% SDS-PAGE and transferred to a PVDF membrane. The membrane was blocked with 5% skim milk and incubated with anti-Flag monoclonal antibody (1:3000, Abmart) overnight at 4°C, followed by HRP-conjugated goat anti-mouse secondary antibody (1:5000). Signals were detected using an ECL chemiluminescence kit (Biyuntian).

### Determination of physiological changes

Following the methods described by Campos *et al* [66] electrolyte leakage rate. DAB and NBT staining were carried out according to Zang *et al* [67]. POD, SOD, MDA, and CAT activities, as well as H_2_O_2_ and proline contents, were measured utilizing assay kits from Nanjing Jianjian (Nanjing Jianjian, Jiangsu, China). For Chl content determination, samples were ground in liquid nitrogen in a 2-mL centrifuge tube, and 2 mL of 50% acetone solution was added. The mixture was incubated in the dark until the material turned white. The optical density was then measured at 663, 645, 652, and 470 nm, using 50% acetone as the blank. Chl content was calculated using the standard formula. The determination of MeJA content refers to the method of the reagent kit (Shanghai COIBO BIO, CB10073). Each sample included at least 10 plantlets, with three biological replicates per experiment.

### EMSA

The CDS of *BpERF1A* was cloned into the pMAL-c5x expression vector to generate an MBP-tagged fusion protein. The constructed plasmid was then transformed into competent *Escherichia coli* ER2523 cells. Protein expression was induced by the addition of 0.2 mM isopropyl-β-d-thiogalactopyranoside (IPTG). The expressed fusion proteins were purified using affinity chromatography with amylose resin, which specifically binds to the maltose-binding protein (MBP) tag. Probes corresponding to G-box elements (Biotin or Biotin-mutant) were synthesized and labeled with biotin at the 5′ end (Shenggong, Shanghai). For competition assays, an unlabeled probes was added to the reaction mixture prior to adding the labeled probe. EMSA reactions were performed using a chemiluminescence EMSA kit (Biyuntian, GS009) according to the manufacturer's instructions. Protein–DNA complexes were resolved on 6% native polyacrylamide gels, transferred to nylon membranes, and detected with streptavidin–HRP chemiluminescence (Tanon-5200) [68].

### Y1H

*BpDHN* and *BpAOS* promoter fragments were amplified by PCR and inserted into the pHIS2 vector. *BpERF1A* was ligated into the pGADT7-rec2 vector to generate a pray plasmid expressing *BpERF1A*-AD. *BpERF1A*-AD and *BpDHN*–pHIS2 or *BpAOS*–pHIS2 were individually transformed into Y187 yeast cells using a yeast transformation kit. The transformed yeast cells were initially grown on SD/-Trp/-Leu medium at 30°C for 3 days. Subsequently, the transformants were inoculated onto SD/-Trp/-Leu/-His medium supplemented with 3-AT and incubated at 30°C for 3 to 5 days for screening.

### ChIP analysis

Using *BpERF1A*-OE plants as material, protein–DNA interactions were crosslinked with 1% (w/v) formaldehyde, following the previously described protocol. The crosslinked chromatin was then fragmented into small pieces using a Covaris sonicator (10% output power, 3 seconds on, 10 seconds off, total 20 minutes) [69]. Immunoprecipitation was performed with a Flag antibody (Abmart, M20008) to enrich DNA fragments bound to *BpERF1A*. After washing and elution, proteins were removed with Proteinase K, and DNA was purified via phenol–chloroform extraction, which was then followed by the ethanol precipitation. Finally, specific target gene fragments were amplified by PCR using the purified DNA as a template.

### BiFC assay

The CDSs of candidate proteins *BpERF1A* and *BpRAV1* were cloned into the N-terminal and C-terminal ends of YFP, respectively. The resulting constructs, *BpERF1A*–YFPN/RAV1–YFPC and the control vectors YFPN/RAV1–YFPC, were transiently introduced into tobacco leaves via *A. tumefaciens* EHA105. Following transformation, the leaves were incubated in the dark at 25°C for 48 hours and subsequently observed using a confocal microscope (Carl Zeiss, Oberkochen, Germany) with an excitation wavelength of 514 nm and an emission range of 527 to 550 nm (YFP channel), and the nucleus was visualized using DAPI staining.

### Complementary luciferase assay

For the luciferase complementation assay in tobacco, the *BpERF1A* CDS was cloned into the KpnI and SalI sites of the pCAMBIA1300–nLUC vector, while the full-length *BpRAV1* sequence was cloned into the KpnI and SalI sites of the pCAMBIA1300–cLUC vector. Agrobacterium GV3101 cultures (OD_600_ = 0.8) carrying these constructs were infiltrated into *tobacco* leaves [70]. The bacterial mixtures were co-infiltrated into *N. benthamiana* leaves, which were then analyzed after a 48-hour incubation. For quantification, LUC activity was measured with a dual-luciferase assay kit (Beyotime, Shanghai, China), and the relative LUC activity was calculated as the ratio of firefly LUC to Renilla LUC (REN) luminescence (LUC/REN). The resulting luminescence was imaged using a Tanon 5200 system (Tanon, Shanghai, China). Each experiment included at least six independent biological replicates. Statistical significance was determined using a two-tailed Student's *t*-test.

### Statistical analysis

All experiments were performed with at least three biological replicates. Data are presented as mean ± standard deviation. Statistical analyses were conducted using GraphPad Prism 10.0. For comparisons between two groups, Student's *t*-test was used. For multiple group comparisons, one-way analysis of variance followed by Tukey's *post hoc* test was applied. Differences were considered statistically significant at *P* < 0.05, with asterisks denoting significance levels as follows: ^*^P < 0.05, ^**^P < 0.01, ^***^P < 0.001, ^****^P < 0.0001.

Primers used in these assays are listed in Table S1.

## Supplementary Material

Web_Material_uhag133

## Data Availability

The data underlying this article are available in the article and in its online supplementary material.
