# Supplementary material for: The transcription factor BpERF1A enhances drought tolerance in Betula platyphylla by activating dehydrin and ROS scavenging through interaction with BpRAV1
Source: Hortic Res. 2026 Apr 7;13(8):uhag133. doi: 10.1093/hr/uhag133 (PMC13437086; doi:10.1093/hr/uhag133)
Supplement: Supplementary file 1 [file web_material_uhag133.zip › Figure S10.pdf]

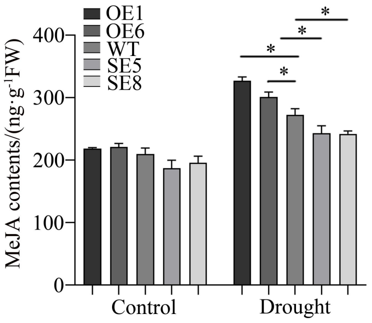
MeJA contents/(ng·g-1FW)
400
300
200
100
0
OE1
OE6
WT
SE5
SE8
*
*
*
*
Control
Drought

Figure S10.
