# Supplementary material for: The transcription factor BpERF1A enhances drought tolerance in Betula platyphylla by activating dehydrin and ROS scavenging through interaction with BpRAV1
Source: Hortic Res. 2026 Apr 7;13(8):uhag133. doi: 10.1093/hr/uhag133 (PMC13437086; doi:10.1093/hr/uhag133)
Supplement: Supplementary file 1 [file web_material_uhag133.zip › Figure S2..pdf]

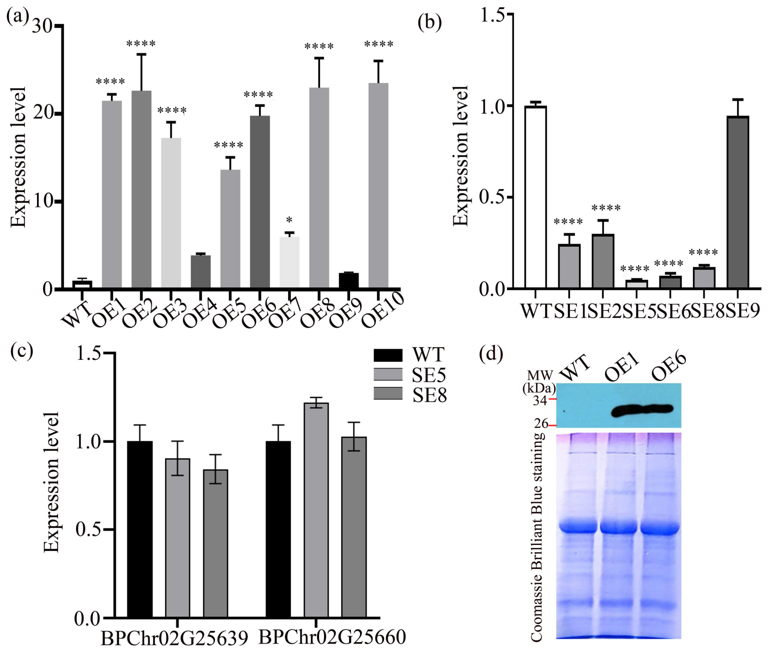

(a)
Expression level
WT
OE1
OE2
OE3
OE4
OE5
OE6
OE7
OE8
OE9
OE10
(b)
Expression level
WT
SE1
SE2
SE5
SE6
SE8
SE9
(c)
Expression level
WT
SE5
SE8
BPChr02G25639
BPChr02G25660
(d)
MW (kDa)
WT
OE1
OE6
34
26
Coomassie Brilliant Blue staining

Figure S2.
