# Supplementary material for: The transcription factor BpERF1A enhances drought tolerance in Betula platyphylla by activating dehydrin and ROS scavenging through interaction with BpRAV1
Source: Hortic Res. 2026 Apr 7;13(8):uhag133. doi: 10.1093/hr/uhag133 (PMC13437086; doi:10.1093/hr/uhag133)
Supplement: Supplementary file 1 [file web_material_uhag133.zip › Figure S3..pdf]

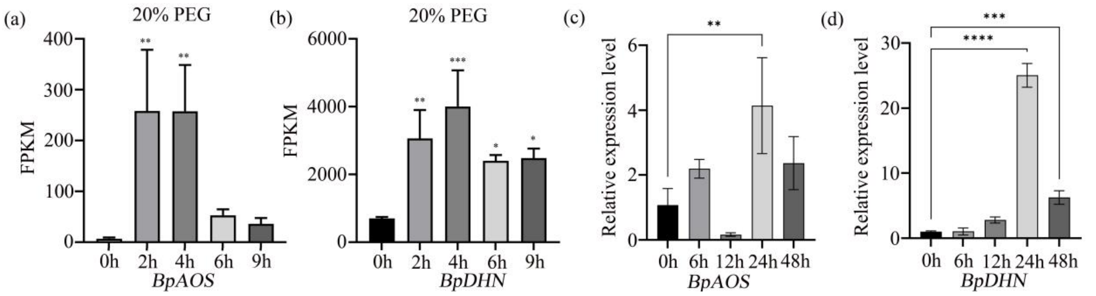
(a)
20% PEG
400
300
200
100
0
FPKM
**
**
0h 2h 4h 6h 9h
BpAOS
(b)
20% PEG
6000
4000
2000
0
FPKM
**
***
*
*
0h 2h 4h 6h 9h
BpDHN
(c)
Relative expression level
6
4
2
0
**
0h 6h 12h 24h 48h
BpAOS
(d)
Relative expression level
30
20
10
0
***
****
0h 6h 12h 24h 48h
BpDHN

Figure S3.
