# Supplementary material for: The transcription factor BpERF1A enhances drought tolerance in Betula platyphylla by activating dehydrin and ROS scavenging through interaction with BpRAV1
Source: Hortic Res. 2026 Apr 7;13(8):uhag133. doi: 10.1093/hr/uhag133 (PMC13437086; doi:10.1093/hr/uhag133)
Supplement: Supplementary file 1 [file web_material_uhag133.zip › Figure S4..pdf]

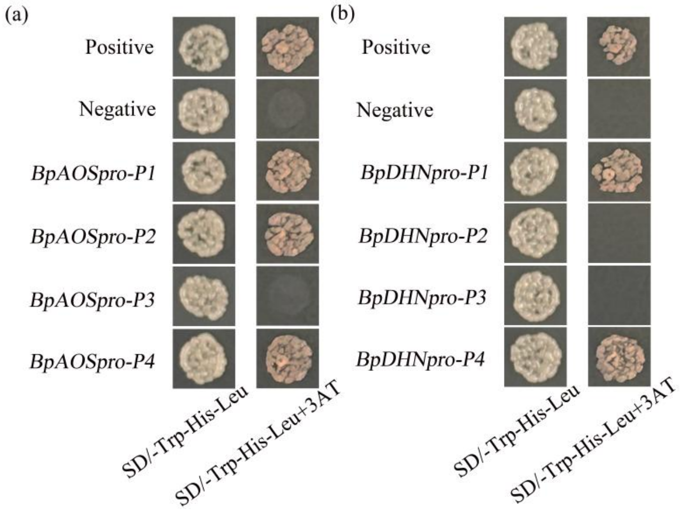
(a)
Positive
Negative
BpAOSpro-P1
BpAOSpro-P2
BpAOSpro-P3
BpAOSpro-P4
SD/-Trp-His-Leu
SD/-Trp-His-Leu+3AT
(b)
Positive
Negative
BpDHNpro-P1
BpDHNpro-P2
BpDHNpro-P3
BpDHNpro-P4
SD/-Trp-His-Leu
SD/-Trp-His-Leu+3AT

Figure S4.
