# Supplementary material for: The transcription factor BpERF1A enhances drought tolerance in Betula platyphylla by activating dehydrin and ROS scavenging through interaction with BpRAV1
Source: Hortic Res. 2026 Apr 7;13(8):uhag133. doi: 10.1093/hr/uhag133 (PMC13437086; doi:10.1093/hr/uhag133)
Supplement: Supplementary file 1 [file web_material_uhag133.zip › Figure S5.pdf]

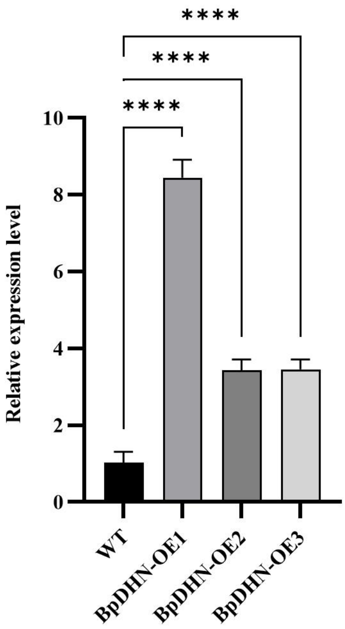

****
****
****
10
8
6
4
2
0
Relative expression level
WT
BpDHN-OE1
BpDHN-OE2
BpDHN-OE3

Figure S5.
