# Supplementary material for: The transcription factor BpERF1A enhances drought tolerance in Betula platyphylla by activating dehydrin and ROS scavenging through interaction with BpRAV1
Source: Hortic Res. 2026 Apr 7;13(8):uhag133. doi: 10.1093/hr/uhag133 (PMC13437086; doi:10.1093/hr/uhag133)
Supplement: Supplementary file 1 [file web_material_uhag133.zip › Figure S7..pdf]

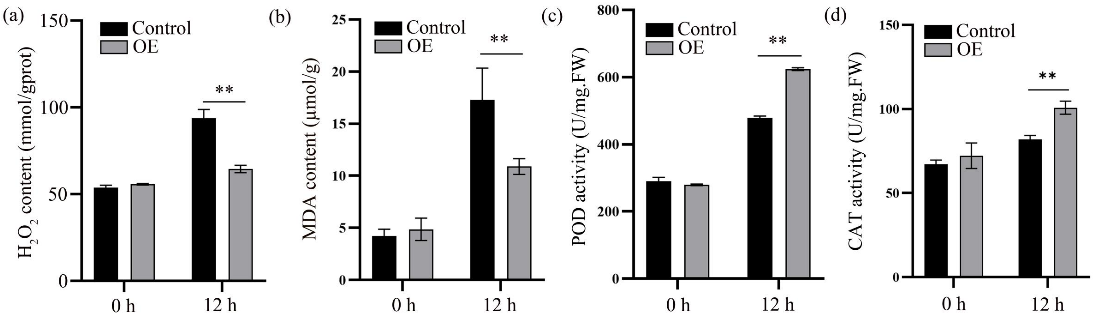
(a)
150
100
50
0
H_2O_2 content (mmol/gprot)
Control
OE
**
0 h
12 h
(b)
25
20
15
10
5
0
MDA content (μmol/g)
Control
OE
**
0 h
12 h
(c)
800
600
400
200
0
POD activity (U/mg.FW)
Control
OE
**
0 h
12 h
(d)
150
100
50
0
CAT activity (U/mg.FW)
Control
OE
**
0 h
12 h

Figure S7.
