# Supplementary material for: The transcription factor BpERF1A enhances drought tolerance in Betula platyphylla by activating dehydrin and ROS scavenging through interaction with BpRAV1
Source: Hortic Res. 2026 Apr 7;13(8):uhag133. doi: 10.1093/hr/uhag133 (PMC13437086; doi:10.1093/hr/uhag133)
Supplement: Supplementary file 1 [file web_material_uhag133.zip › Figure S8..pdf]

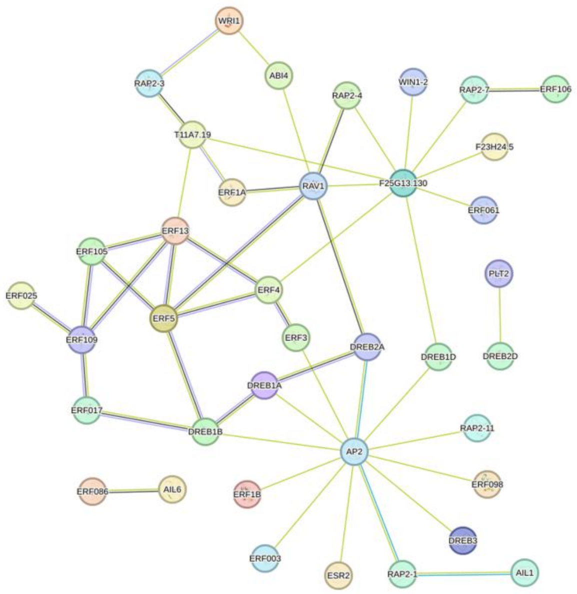
WRI1
RAP2-3
ABI4
RAP2-4
WIN1-2
RAP2-7
ERF106
T11A7.19
F23H24.5
ERF1A
RAV1
F25G13.130
ERF061
ERF13
ERF105
PLT2
ERF025
ERF4
ERF5
ERF109
ERF3
DREB2A
DREB1D
DREB2D
DREB1A
ERF017
DREB1B
RAP2-11
AP2
ERF086
AIL6
ERF1B
ERF098
DREB3
ERF003
ESR2
RAP2-1
AIL1

Figure S8.
