# Supplementary material for: The transcription factor BpERF1A enhances drought tolerance in Betula platyphylla by activating dehydrin and ROS scavenging through interaction with BpRAV1
Source: Hortic Res. 2026 Apr 7;13(8):uhag133. doi: 10.1093/hr/uhag133 (PMC13437086; doi:10.1093/hr/uhag133)
Supplement: Supplementary file 1 [file web_material_uhag133.zip › Supplementary Figure.docx]

Figure S1. Expression of the *BpERF1A* transcription factor in the drought-stressed transcriptome.

Figure S2. Expression of BpERF1A in WT and transgenic brich.

(a) qRT-PCR analysis of the relative expression of BpERF1A in the OE lines. (b) qRT-PCR analysis of the relative expression of BpERF1A in the SE lines. (c) qRT-PCR analysis of the relative expression levels of the homologous genes of *BpERF1A* in SE lines. (d) Analysis ofthe BpERF1A protein levels in wild-type (WT) and OE. Data are presented as the mean ± SD (n ≥ 3). Asterisks denote significant differences versus the control (one-way ANOVA with Tukey’s test). (**P* < 0.05, ***P* < 0.01, ****P*< 0.001, *****P*< 0.0001).

Figure S3. Analysis of *BpAOS* and *BpDHN* gene expression under PEG_6000_ stress conditions. (a) Expression of the *BpAOS* in the drought-stressed transcriptome. (b) Expression of the *BpDHN* in the drought-stressed transcriptome. Expression patterns of *BpAOS* (c) and *BpDHN* (d) in 20% PEG_6000_ treatment. Data are presented as the mean ± SD (n ≥ 3). Asterisks denote significant differences versus the control (one-way ANOVA with Tukey’s test) (**P* < 0.05, ***P* < 0.01, ****P*< 0.001, *****P*< 0.0001).

.

Figure S4. BpERF1A directly regulates *BpAOS* and *BpDHN* transcription by binding to their promoters.

(a) Y1H assay confirming BpERF1A binding to the promoters of *BpAOS* (P1-P4, p1:-552bp-325bp, -552bp-628bp, -848bp-628bp, -1425bp-1208bp). (b) Y1H assay confirming BpERF1A binding to the promoters of *BpDHN* (P1-P4, p1:-311bp-90bp, -611bp-384bp, -910bp-686bp, -1357bp-1135bp). Positive control: p53-HIS2/pGADT7-Rec2-p53; negative control: pGADT7-Rec2-*BpERF1A*/p53-HIS2. 3AT: 50 mM.

Figure S5. qRT-PCR analysis of the relative expression of BpDHN in the OE lines.

Figure S6. *BpAOS* **transcript levels in birch plants following transient overexpression.** One-month-old birch plants were transiently transformed with the empty vector pROKII (Control) or with 35S::BpAOS. Plants were subsequently grown on 1/2 MS medium for 24 h, after which the expression level of BpAOS was measured in whole seedlings by qRT-PCR. Error bars represent the standard deviation from multiple technical replicates. OE, BpAOS overexpression; Con, pROKII empty vector control.

Figure S7. Physiological analysis of the WT and OE lines. (a) H_2_O_2_ content . (b) MDA content. (c) POD activity. (d) CAT activity. Data are presented as the mean ± SD (n ≥ 3). Asterisks denote significant differences versus the control (one-way ANOVA with Tukey’s test)(**P* < 0.05, ***P* < 0.01, ****P*< 0.001, *****P*< 0.0001).

Figure S8. Prediction of AP2/ERF family member interaction proteins.

Figure S9. Analysis of *BpRAV1* gene expression under PEG_6000_ stress conditions.

Figure S10. MeJA contents.
